# Ago2-Mediated Recruitment of HP1a on Transposable Elements in *Drosophila* Brain

**DOI:** 10.3390/cells14171361

**Published:** 2025-09-01

**Authors:** Oxana M. Olenkina, Ruslan A. Simonov, Anna Y. Ivannikova, Yuri A. Abramov, Anastasiia L. Sivkina, Sergey V. Ulianov, Yuri Y. Shevelyov

**Affiliations:** 1 Laboratory of Analysis of Gene Regulation, National Research Centre “Kurchatov Institute”, Moscow 123182, Russia; oxamyth@gmail.com (O.M.O.); simonoff.ra@mail.ru (R.A.S.); ya-annushka@mail.ru (A.Y.I.); abramov75@rambler.ru (Y.A.A.); 2 Department of Cellular Genomics, Institute of Gene Biology, Russian Academy of Sciences, Moscow 119334, Russia; anastasiia.sivkina@gmail.com (A.L.S.); sergey.v.ulyanov@gmail.com (S.V.U.)

**Keywords:** Argonaute 2 (Ago2), heterochromatin protein 1a (HP1a), lamin Dm0, short interfering RNAs (siRNAs), transposable element, brain, *Drosophila*

## Abstract

In *Drosophila* gonads, transposable elements (TEs) are repressed by the Piwi-interacting RNA (piRNA) pathway operating both co-transcriptionally and post-transcriptionally. In the non-gonadal tissues, TEs are mainly repressed by the short interfering RNA (siRNA) pathway with Argonaute 2 (Ago2) functioning as an effector protein. It is generally assumed that this pathway acts at the post-transcriptional level. However, recent data point to its possible involvement in co-transcriptional silencing as well. Here, using DamID, we found a drastic decrease in HP1a on TEs (especially on the LTR-containing retrotransposons) and other heterochromatin regions in *Ago2*-mutant *Drosophila* brain. HP1a reduction is accompanied by the increased chromatin accessibility of TEs, indicating their derepression. Accordingly, several LTR-containing retrotransposons were up-regulated in the larval brain of *Ago2* mutants. Moreover, upon the knock-down of lamin *Dm0* in neurons, HP1a was increased predominantly on the same set of TEs that had reduced HP1a binding in *Ago2* mutants. We hypothesize that, since Ago2 was localized to the common complex with lamin Dm0, the depletion of the latter may release Ago2 in the nucleoplasm, thus enhancing the recruitment of HP1a on TEs. Our findings support the hypothesis that TEs in the *Drosophila* brain are silenced, in part, through Ago2-mediated recruitment of HP1a.

## 1. Introduction

The cytological term “heterochromatin” was originally proposed to designate the darkly stained regions of chromosomes, implying their denser structure compared to euchromatin. Facultative heterochromatin (fHet) is interspersed with euchromatin along the entire length of chromosomes, with the exception of pericentromeric and telomeric regions where constitutive heterochromatin (cHet) resides [1]. fHet is known to contain silent tissue-specific genes and transposable elements (TEs), whereas cHet contains few genes and is mainly composed of TEs and other moderately and highly repetitive sequences. Microscopy observations have shown that heterochromatin is predominantly located at the nuclear periphery and around nucleoli, although in some cell types of mammals, it may aggregate in the center of nucleus [2]. Accordingly, heterochromatin regions strongly overlap with the lamina-associated domains (LADs) [3,4,5,6,7] which represent regions interacting with the nuclear lamina (NL) – a meshwork of lamins and lamin-associated proteins lining the nuclear envelope [8].

Since transpositions of TEs are potentially harmful for any organism, various strategies for TE repression have evolved [9]. In *Drosophila* gonads, TEs are repressed by the Piwi-interacting RNA (piRNA) pathway, which acts at both co-transcriptional and post-transcriptional levels. Co-transcriptional silencing is initiated by the Piwi/piRNA complex, which recognizes nascent TE transcripts and recruits to them, among others, the components of general heterochromatin machinery, such as histone methyltransferase Eggless/SETDB1 introducing H3K9me2/3 histone modifications, H3K4 demethylase Lsd1, and HP1a [10,11,12,13]. In *Drosophila* heads, the main components of the piRNA-silencing system are not expressed [14]. Moreover, in heads and other non-gonadal tissues, TEs are likely repressed by the small interfering RNA (siRNA) pathway [15,16,17]. It was generally assumed that the major mechanism of its action is post-transcriptional silencing, specifically the cleavage of TE transcripts in the cytoplasm, recognized by complementary siRNAs associated with the Argonaute 2 (Ago2) effector protein [18]. However, accumulating evidence suggests that Ago2 in *Drosophila* is also present in the nucleus, where it interacts with chromatin [19,20]. Moreover, *Ago2* mutations were shown to affect chromatin structure [21,22,23,24,25]. In particular, *Ago2* mutations lead to HP1a accumulation in the ectopic sites of *Drosophila* polytene chromosomes [22], as well as to the reduced HP1a binding in both cHet and fHet regions in *Drosophila* heads [25]. However, data on the ability of Ago2 to recruit HP1a to TEs for their repression are controversial [19,25]. Moreover, according to ChIP-seq mapping in S2 cells, Ago2 binding sites strongly colocalize with chromatin insulators in euchromatin and have not been revealed in heterochromatin [20]. Therefore, the model according to which Ago2 is involved in the co-transcriptional silencing of TEs in *Drosophila* somatic cells remains to be fully elucidated.

In this work, we investigated whether Ago2 is involved in the recruitment of HP1a to TEs and other heterochromatin regions in *Drosophila* brain. We also explored whether the depletion in neurons of lamin Dm0, which is the main component of the NL, affects HP1a occupancy on TEs and other repeats. Finally, we examined whether HP1a binding to TEs results in a more compact state of the associated chromatin. Taken together, our results indicate the existence in *Drosophila* brain of an Ago2-mediated silencing mechanism which acts through the recruitment of HP1a at TE chromatin.

## 2. Materials and Methods

### 2.1. Fly Crossing and Transgenic Lines

To perform Dam identification (DamID) with HP1a in the central brain of third instar male larvae, we employed fly lines y^1^ w*; M{w^+mC^ = hs-dam.4-HT-intein-L127C}ZH-51C and y^1^ w*; M{w^+mC^ = hs-dam.4-HT-intein-L127C-HP1}ZH-51C (#65429 and #65432; Bloomington Drosophila Stock Center) carrying *Dam^4-HT-intein@L127C^* or *Dam^4-HT-intein@L127C^-HP1a* constructs under the control of the *hsp70* gene promoter inserted at the *51C* site of chromosome *2* [26,27]. These lines contain an intein insertion in the coding region of Dam methylase, which may be removed by self-splicing after an addition of 4-hydroxytamoxifen (4-HT). However, in some organs, including the central brain of third instar larvae, these constructs yield a methylation level sufficient for DamID without the addition of 4-HT and without heat shock induction of the *hsp70* promoter [26,27].

For *Ago2* mutation, we employed *Ago2^414^/Ago2^454^* trans-heterozygous mutant alleles, where *Ago2^454^* is the null allele [28], whereas *Ago2^414^* was first characterized as a null mutant allele, but further appeared to be the hypomorphic allele [28,29].

For obtaining lamin *Dm0* knock-down (Lam-KD) in neurons, we combined the neuron-specific driver line *P{GAL4-elav.L}3* (former #8760, Bloomington Drosophila Stock Center, Bloomington, IN, USA) with the line *P{KK102399}VIE-260B* carrying the hairpin construct *UAS::ds-laminDm0* (#107419, Vienna Drosophila Resource Center) or the hairpin construct to *white* (*UAS::ds-white*, #33623, Bloomington Drosophila Stock Center) as a control. Next, using conventional genetic crosses, we combined the constructs for HP1a-DamID with the *Ago2*-mutant alleles or with the constructs for Lam-KD.

For performing HP1a-DamID in an Ago2-mutant background, we obtained the following fly genotypes: 1) *Dam^4-HT-intein@L127C^/Dam^4-HT-intein@L127C^; Ago2^414^/Ago2^454^* 2) *Dam^4-HT-intein@L127C^-HP1a/Dam^4-HT-intein@L127C^-HP1a; Ago2^414^/Ago2^454^* 3) *Dam^4-HT-intein@L127C^/Dam^4-HT-intein@L127C^; Ago2^WT^/Ago2^WT^* 4) *Dam^4-HT-intein@L127C^-HP1a/Dam^4-HT-intein@L127C^-HP1a; Ago2^WT^/Ago2^WT^* (the third and the fourth genotypes were used as controls).

For performing HP1a-DamID upon Lam-KD in neurons, we obtained the following fly genotypes: 1) *Dam^4-HT-intein@L127C^/UAS::ds-laminDm0; elav::Gal4/+* 2) *Dam^4-HT-intein@L127C^-HP1a/UAS::ds-laminDm0; elav::Gal4/+* 3) *Dam^4-HT-intein@L127C^/UAS::ds-white; elav::Gal4/+* 4) *Dam^4-HT-intein@L127C^-HP1a/UAS::ds-white; elav::Gal4/+* (the third and the fourth genotypes were used as controls).

### 2.2. DamID with HP1a in Brain

The central brain was extracted in two replicates from approximately one hundred third instar male larvae of the corresponding genotypes. Several micrograms of genomic DNA were isolated from the larval brain. After that, PCR amplification of methylated G^m6^ATC-G^m6^ATC fragments of the genome was carried out according to [30]. Briefly, ~0.5 µg genomic DNA was digested by DpnI (New England Biolabs, Ipswich, MA, USA) at the methylated G^m6^ATC sites, followed by the ligation of PCR adaptors (Supplementary Figure S1a). Next, unmethylated GATC sites within these restriction fragments were digested by DpnII (New England Biolabs, Ipswich, MA, USA). After that, PCR-amplification of the fragments that contained the methylated G^m6^ATC sites at both ends was performed. A total of 19 cycles or 17 cycles of PCR (1 min at 94 °C, 1 min at 65 °C, and 2 min at 68 °C) were applied for Dam and Dam-HP1a samples, respectively, isolated from *Ago2^mut^* and *Ago2^WT^* brain, whereas 19 cycles or 18 cycles of PCR were applied for Dam and Dam-HP1a samples, respectively, isolated from Lam-KD and control brain. After separation by electrophoresis in agarose gel, the amplified fragments produced a characteristic smear in the range of several hundred to several thousand bp. This smear was practically not visible in the control sample, which was not treated with Dpn I (i.e., in the control without cleavage at the methylated sites of genome) (Supplementary Figure S1b,c). These results indicate that PCR amplification of genomic fragments occurred on the fragments which were methylated in vivo by Dam-HP1a, and not on those formed as a result of random breaks in the genomic DNA during its isolation. The discrete bands detected over the smear represented fragments from the mitochondrial genome and were characteristic of the “intein” DamID system [26]. Next, PCR-amplified methylated fragments were digested with DpnII (New England Biolabs, Ipswich, MA, USA) to remove adaptors and were purified using a PCR purification kit (Qiagen, Germantown, MD, USA). Libraries from ~0.5 µg DNA samples were then prepared for next-generation sequencing on Illumina. Sequencing was performed on the Illumina NovaSeq X by the company “Sequentio” (https://sequentio.ru/ (accessed on 30 August 2025)), which yielded from ~55 to ~82 million 150-nt paired-end reads per sample (Supplementary Table S1).

### 2.3. Bioinformatics Analysis of DamID Data

Sequencing reads from two biological replicates of Dam-only and Dam-HP1a samples were adapter clipped with *trim_galore* version 0.6.10 (https://github.com/FelixKrueger/TrimGalore (accessed on 2 February 2023)) and were mapped to the dm3/R5 genome assembly with “*bowtie2*” version 2.5.4 [31] using standard parameters. The DamID profiles on the dm3 genome or on TE consenus sequences were generated with “*deepTools*” version 3.5.4 [32] with either no mapping quality threshold or a threshold set to 40 to exclude non-uniquely mapped reads. Counts per million (CPMs) of Dam-only or Dam-HP1a samples for each replicate were calculated by “*HTSeq-count*” [33], and Dam-HP1a values were normalized to Dam-only values and log_2_-transformed. Since replicates were highly, or, in the case of *Ago2^mut^* replicates 1 and 2, moderately correlated (Supplementary Figure S2), they were combined, and the same procedure was applied for them.

To generate log_2_(Dam-HP1a/Dam) and log_2_(Dam-only) profiles across TEs, sequencing reads from two biological replicates of Dam-only and Dam-HP1a samples were mapped on the canonical TE sequences (https://github.com/bergmanlab/drosophila-transposons/blob/master/current/D_mel_transposon_sequence_set.fa (accessed on 30 August 2021)), which were divided onto 50-bp bins. Reads were counted and converted to CPM by normalization to the number of reads mapped to the dm3 genome. Dam-HP1a values were normalized to Dam-only values and log_2_-transformed. Next, average log_2_(Dam-HP1a/Dam) and average log_2_(Dam-only) values for each TE were calculated (Supplementary Table S2).

Subsequent analysis including plot generation was carried out in *R* (version 4.5.0) and *Rstudio* (version 2025.05.0+496). Scripts for mapping, profile generation, and analysis were deposited in the GitHub repository (https://github.com/LARG-IMG/Dm_brain_AGO2_2025 (accessed on 5 August 2025)).

### 2.4. Immunostaining of *Drosophila* Brain

The central brain from ~50 third instar larvae was immunostained as previously described [34]. A mix of mouse monoclonal anti-Lamin Dm0 (1:500; ADL84 and ADL67 [35]) and rabbit polyclonal anti-HP1a (1:500; BioLegend, San Diego, CA, USA) antibodies was applied. As the secondary, Alexa Fluor 546-conjugated goat anti-rabbit IgG (Invitrogen, Waltham, MA, USA) or Alexa Fluor 633-conjugated goat anti-mouse IgG (Invitrogen, Waltham, MA, USA) antibodies were applied.

### 2.5. RT-qPCR Analysis

Total RNA was isolated from approximately one hundred central brain of third instar male larvae using Trizol reagent (Invitrogen, Waltham, MA, USA), and contaminating DNA was removed by DNase I treatment followed by DNase I removal by the DNAse Inactivation Reagent (Ambion, Waltham, MA, USA). A real-time RT-qPCR assay for selected TEs was performed in 3 biological replicates on cDNAs synthesized with random primers using SuperScript II reverse transcriptase (Invitrogen). RT-qPCR analysis was performed on the LightCycler 96 Instrument (Roche, Basel, Switzerland) using SYBR Green chemistry (Evrogen, Moscow, Russia), and primer pairs are provided in Supplementary Table S3. Data were normalized to *Actin5C* gene expression.

### 2.6. Statistical Analysis

For *p*-value estimation, the Mann–Whitney *U* test was used for the comparison of two sample distributions.

## 3. Results

### 3.1. HP1a Is Drastically Decreased on TEs and in cHet Regions in Ago2-Mutant *Drosophila* Brain

To test whether Ago2 represses TEs by recruiting to them HP1a, we performed DamID with HP1a in *Drosophila* central brain from third instar larvae carrying *Ago2^414^/Ago2^454^* trans-heterozygous mutant alleles (hereinafter, the *Ago2*-mutant line) and in the control wild-type (WT) line. *Ago2^454^* is the null allele [28], whereas *Ago2^414^* was first characterized as a null allele, but further appeared to be the hypomorphic allele [28,29]. RT-qPCR analysis using a primer pair for the fifth exon of *Ago2* gene revealed a 4.5-fold down-regulation of *Ago2* expression in the larval brain from *Ago2^414^/Ago2^454^* trans-heterozygous mutant alleles relative to *Ago2^WT^* (Supplementary Figure S3). By immunostaining, we did not detect noticeable changes in the localization of HP1a in larval brain nuclei in the *Ago2*-mutant background. The pericentromeric heterochromatin, revealed by bright HP1a staining, is localized mainly in close proximity to the NL in both control and *Ago2*-mutant brain (Supplementary Figure S4a).

To perform DamID, we employed the “intein” DamID system [26,27], which works well in the larval central brain without 4-hydroxytamoxifen or heat shock induction [26]. Using genetic crosses, we obtained flies carrying constructs for DamID combined with *Ago2^414^/Ago2^454^* alleles or with the WT allele (as a control). After the isolation of genomic DNA from the central brain, we performed PCR-amplification of methylated genomic fragments (Supplementary Figure S1a,b), followed by the sequencing of libraries on Illumina and the construction of two replicates of log_2_(Dam-HP1a/Dam) profiles in the *Ago2*-mutant and in the WT larval central brain.

**Figure 1:**
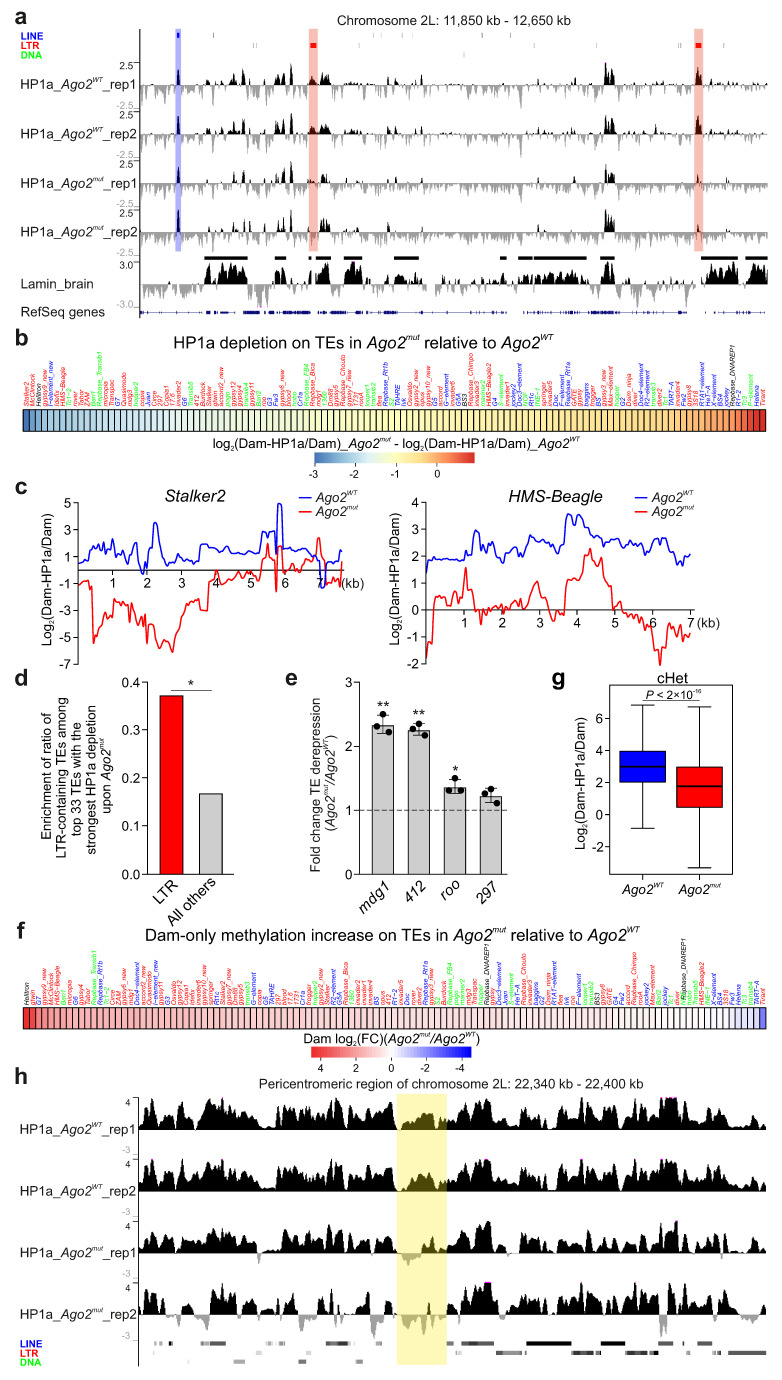
HP1a is drastically decreased on TEs and in cHet regions in *Ago2*-mutant *Drosophila* brain. (**a**) A screenshot of UCSC Genome Browser showing the log_2_(Dam-HP1a/Dam) profiles for *Ago2^WT^* (two replicates) and *Ago2^mut^* (two replicates) region of the 2L chromosome. RepeatMasker over profiles indicates LINE TEs (blue), LTR-containing TEs (red), and DNA transposons (green). Below are the lamin Dm0 profile in the larval central brain [27], with LADs shown as black rectangles over the profile, and RefSeq genes. The red translucent rectangles highlight two LTR-containing TEs in the region which had reduced HP1a binding upon *Ago2* mutation, while the blue translucent rectangle shows a LINE element without any HP1a decrease. (**b**) Heatmap showing the difference between average log_2_(Dam-HP1a/Dam) values on the canonical TE copies in *Ago2*-mutant relative to WT larval central brain. (**c**) HP1a profiles across two LTR-containing TEs in *Ago2*-mutant (red) and WT (blue) larval central brain. (**d**) Ratio of LTR-containing TEs among all TEs and ratio of all other TEs among all TEs for the 33 TEs with the strongest reduction in HP1a binding in the *Ago2*-mutant background. *p*-value < 0.05 (*), Z-test. (**e**) RT-qPCR analysis of LTR-containing TE expression in *Ago2*-mutant and WT larval central brain. Data of three replicates were normalized to *Actin5C* gene expression. *p*-value < 0.05 (*); *p*-value < 0.01 (**); one-sample two-tailed *t*-test. (**f**) Heatmap showing log_2_(fold change) of average Dam-only methylation on the canonical TE copies in *Ago2*-mutant relative to WT larval central brain. (**g**) Box-plots showing log_2_(Dam-HP1a/Dam) values in cHet regions in *Ago2*-mutant (red) and WT (blue) larval central brain. *p*-value was determined via a Mann–Whitney *U*-test. (**h**) A screenshot of UCSC Genome Browser showing the log_2_(Dam-HP1a/Dam) profiles for *Ago2^WT^* (two replicates) and *Ago2^mut^* (two replicates) pericentromeric region of 2L chromosome. RepeatMasker below profiles indicates LINE TEs (blue), LTR-containing TEs (red), and DNA transposons (green). The yellow translucent rectangle highlights a region lacking TEs but nevertheless showing a reduction in HP1a in the *Ago2*-mutant background.

Visual examination of HP1a profiles built upon non-unique mapping of sequencing reads indicates that, in the *Ago2*-mutant line, HP1a peaks were drastically decreased on some TE copies compared to those in the WT (Figure 1a). For an unknown reason, this decrease was more pronounced in the second replicate than in the first one. The clustering of DamID replicates showed that WT replicates were highly correlated, while *Ago2*-mutant replicates were only moderately correlated (Supplementary Figure S2a). Nevertheless, since we found the decrease in HP1a on TEs in both *Ago2*-mutant replicates, albeit to different extent (Figure 1a), we combined the replicates for the downstream analysis. Using the combined replicates, we built average HP1a profiles across the canonical TE sequences representing different TE families. Importantly, we found that the significant reduction in HP1a binding in the *Ago2*-mutant background was predominantly observed at the long terminal repeat (LTR)-containing retrotransposons, which are overrepresented among other TE families by this criterion (Figure 1b,c,d and Supplementary Table S2).

Next, we assayed by RT-qPCR the fold change of TE derepression in the *Ago2*-mutant background compared to the WT in the central brain of third instar larvae for several LTR-containing retrotransposons that were previously shown to be derepressed in the heads of imago *Ago2*-mutant flies [16]. Similarly to what was observed in the work of Ghildiyal et al. [16], we found small but significant derepression of the analyzed LTR-containing TEs *mdg1*, *412*, and *roo* (Figure 1e).

Numerous data indicate that HP1 may repress transcription [36,37,38], likely through chromatin condensation [39,40,41]. In *Drosophila* neurons, genes located in LADs bound with HP1a were shown to have lower expression levels than genes in LADs not bound with it [27], thus indicating HP1a-mediated repression. We then explored whether HP1a binding with TEs in the larval brain makes their chromatin more condensed. We took advantage of the fact that Dam-only protein methylates chromatin regions according to their accessibility [42,43]. We built average Dam-only methylation profiles (in CPM) across TEs in WT and in *Ago2*-mutant brain. One can see that Dam-only methylation was increased on the majority of TEs in the *Ago2*-mutant background (Figure 1f), thus indicating that their chromatin became more accessible. Therefore, HP1a recruitment on TEs via Ago2 makes their chromatin more condensed, thus leading to repression.

Next, we divided cHet regions of *Drosophila* genome, determined according to [44], into bins and calculated the fold change of log_2_(Dam-HP1a/Dam) values per bin in the *Ago2*-mutant relative to the WT. As expected, we found a pronounced decrease in HP1a in the cHet regions in *Ago2*-mutant brain compared to WT brain (Figure 1g). We note, however, that the reduction in HP1a binding in cHet was detected not only on the TEs, but also in the regions devoid of TEs (Figure 1h). We conclude that Ago2 recruits HP1a on TEs, as well as on other cHet repeats.

### 3.2. HP1a Is Mainly Increased on the LTR-Containing Retrotransposons and in cHet Regions upon Lam-KD in Neurons

Since Ago2 was shown to be present in the common complex with the lamin Dm0 [45], we examined the participation of lamin Dm0 in the recruitment of HP1a on TEs in the brain. Using the “intein” DamID system [26,27], we performed DamID with HP1a in the central brain from third instar larvae of control flies and flies with Lam-KD in neurons. Although the lamin Dm0 depletion in neurons was severe, it did not lead to notable perturbations of HP1a distribution in the nuclei (Supplementary Figure S4b).

We obtained flies carrying constructs for DamID combined with *UAS::ds-laminDm0* and *elav::Gal4* transgenes, or with control *UAS::ds-white* and *elav::Gal4* transgenes. After isolation of genomic DNA from larval central brain, PCR-amplification of methylated genomic fragments (Supplementary Figure S1c) and sequencing of libraries on Illumina, we constructed log_2_(Dam-HP1a/Dam) profiles for two replicates in control and Lam-KD flies. Since the replicates were highly correlated (Supplementary Figure S2b), we merged them for further analysis.

**Figure 2:**
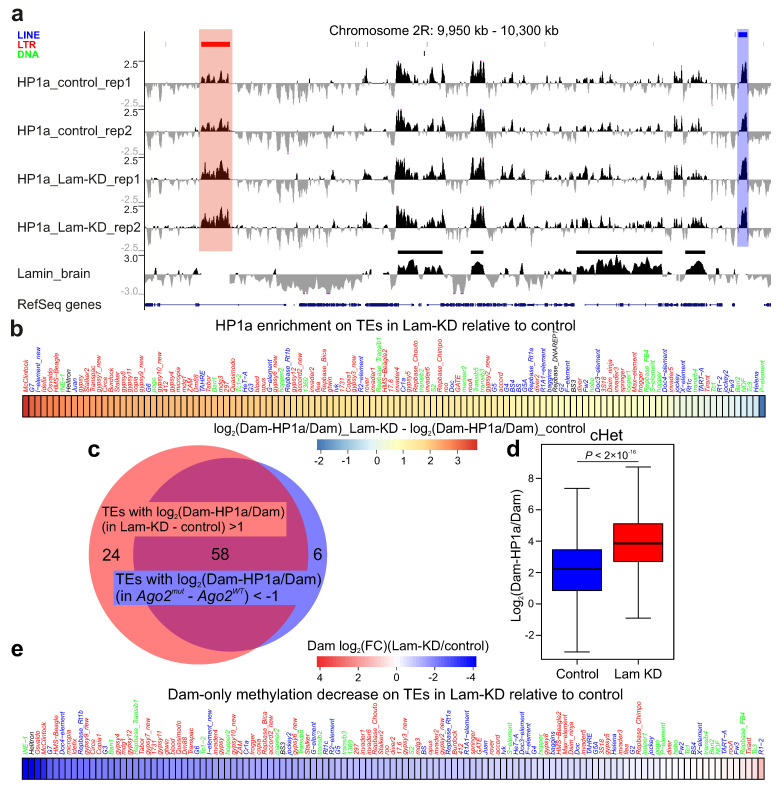
HP1a is increased on TEs and in cHet regions upon Lam-KD in *Drosophila* brain. (**a**) A screenshot of UCSC Genome Browser showing the log_2_(Dam-HP1a/Dam) profiles in a region of the 2L chromosome in the larval central brain with Lam-KD in neurons (two replicates) and in control larval central brain (two replicates). RepeatMasker over profiles indicates LINE TEs (blue), LTR-containing TEs (red), and DNA transposons (green). Below are the lamin Dm0 profile in larval central brain [27], with LADs shown as black rectangles over the profile, and RefSeq genes. The red translucent rectangle highlights a LTR-containing TE in the region which has increased HP1a binding upon Lam-KD, while the blue translucent rectangle shows a LINE element without any HP1a increase. (**b**) Heatmap showing the difference between average log_2_(Dam-HP1a/Dam) values on the canonical TE copies upon Lam-KD relative to the control in larval central brain. (**c**) Venn diagram showing the degree of overlap between TE sets, either having average log_2_(Dam-HP1a/Dam) values in Lam-KD relative to the control >1, or having average log_2_(Dam-HP1a/Dam) values in *Ago2^mut^* relative to *Ago2^WT^* <−1. (**d**) Box-plots showing log_2_(Dam-HP1a/Dam) values in cHet regions upon Lam-KD in neurons (red) and in the control (blue) larval central brain. *p*-value was determined via a Mann–Whitney *U*-test. (**e**) Heatmap showing log_2_(fold change) of average Dam-only methylation on the canonical TE copies in Lam-KD relative to control larval central brain.

Visual examination of the HP1a profile has shown that, upon Lam-KD, HP1a peaks were notably increased in the genomic regions mainly carrying LTR-containing retrotransposons (Figure 2a). The average HP1a profiles across the canonical TEs upon Lam-KD in neurons confirmed a significant increase in HP1a binding at numerous TEs, predominantly at those containing LTRs (Figure 2b). Moreover, the sets of TEs with highly increased or decreased HP1a levels upon Lam-KD or *Ago2* mutation, respectively, strongly overlapped (Figure 2c). We conclude that Lam-KD in neurons enhances HP1a binding with the same TE set which has reduced HP1a binding in the *Ago2*-mutant background.

Next, we calculated log_2_(Dam-HP1a/Dam) values for each genomic bin and compared these distributions in cHet regions in the control and Lam-KD central brain. Upon Lam-KD, HP1a binding appears to be enhanced in the cHet regions (Figure 2d). Finally, Dam-only read mapping indicates that, upon Lam-KD, the accessibility of chromatin on TEs is decreased (Figure 2e), thus indicating a more compact state of their chromatin.

## 4. Discussion

The results of our study together with the data from the literature indicate the existence in *Drosophila* somatic non-gonadal tissues of TE silencing mechanism which functions through the Ago2-mediated recruitment of HP1a at TE chromatin. Several lines of evidence support this model. (i) Upon *Ago2* mutation, HP1a in *Drosophila* brain is drastically decreased on TEs, predominantly on the LTR-containing retrotransposons (Figure 1a–c). These data indicate that HP1a recruitment on TEs is the Ago2-dependent process. (ii) Several LTR-containing TEs are derepressed in *Ago2*-mutant brain (Figure 1e and [16]), thus indicating their silencing with the participation of Ago2. (iii) The up-regulated level of Dam-only methylation on TEs upon *Ago2* mutation (Figure 1f) suggests that the recruitment of HP1a on TEs makes their chromatin less accessible. These results point to a mechanism of TE repression which is based on chromatin condensation. (iv) Lam-KD in *Drosophila* neurons results in the enhanced binding of HP1a mainly with the same TEs which have the reduced HP1a level upon *Ago2* mutation (Figure 2a–c). Because lamin Dm0 and Ago2 physically associate with each other [45], it is reasonable to suggest that Lam-KD may release Ago2 molecules from the association with the NL and Ago2 would be able to more efficiently recruit HP1a on TEs. We note, however, that, in contrast to our observation in the larval central brain, Lam-KD in the fat body of adult flies results in the reduced H3K9me3 level and in TE derepression [46], which may be explained by organ- and/or stage-specific differences.

Since Ago2 was shown to interact with the insulator proteins CP190 and CTCF as well as with RNA Polymerase II [20,23,45], we cannot formally exclude the possibility that HP1a recruitment on TEs in *Drosophila* brain may occur without the recognition of nascent TE transcripts by complementary siRNAs. However, studies on other organisms support the hypothesis that HP1a recruitment on TEs is likely mediated by the Ago2/siRNA complex. Fission yeast *S*. *pombe* was the first model organism where siRNA-dependent heterochromatin formation was described [47,48,49]. Later reports have shown that the siRNA pathway is involved in the establishment of heterochromatin in *C. elegans* [50,51], *A. thaliana* [52,53], and mammals [54,55,56]. For example, a recent study has shown that Ago2 co-transcriptionally represses TEs in quiescent mammalian cells [57]. It should be mentioned, however, that this silencing is supposed to occur not by the deposition of H3K9me3 heterochromatin mark on TEs (which is not changed upon *Ago2* knock-down), but by Ago2-mediated cleavage of the nascent TE transcripts [57]. In *Drosophila*, the ectopic expression of siRNAs, directed against the 1.688^X^ satellite repeats, affects the deposition of H3K9me2 heterochromatin mark on the adjoining chromatin regions [58]. Furthermore, according to chromatin RNA immunoprecipitation data, Ago2 in *Drosophila* may be bound to the nascent transcripts of the expressed genes [59]. Finally, the overrepresentation of siRNAs to the LTR-containing TEs in *Drosophila* heads [16], consistent with the marked reduction in HP1a binding predominantly on the same TE class in brain upon *Ago2* mutation (Figure 1d), is also in line with the HP1a recruitment on TEs through the Ago2/siRNA complex. Abundant siRNAs derived from the LTR-containing retrotransposons may arise as a result of active sense and antisense transcription of these TEs [60,61].

It remains unclear how Ago2 can repress TEs and other repeats if it is only weakly associated with these genome regions. By using ChIP-seq, Ago2 binding sites were previously mapped genome-wide in *Drosophila* S2 cells [20] and, later, in the brain [25]. Moshkovich et al. [20] have found that binding sites of Ago2 strongly colocalized with the insulators in euchromatin, but not with TEs and other repeats in heterochromatin. Lee et al. [25] have revealed Ago2 binding sites in the pericentromeric heterochromatin, but they only poorly colocalized with the sites where HP1a was reduced in the *Ago2*-mutant background. An almost complete absence of Ago2 binding sites on TEs and in the pericentromeric heterochromatin regions is reminiscent of what was observed for Piwi, the key effector protein of the piRNA-silencing pathway. It was shown by both ChIP-seq and DamID that Piwi is not significantly associated with DNA of TEs [34,62]. However, Piwi is transiently associated with nascent TE transcripts via complementarity with the loaded piRNAs, which leads to the recruitment of various components of silencing machinery, such as HP1a, Eggless/SETDB1, etc., on TE chromatin [10,11,13]. Thus, like Piwi/piRNA, the Ago2/siRNA complex may only trigger the co-transcriptional silencing mechanism of TEs and other repeats but is not required for its maintenance.

What components of the Ago2 complex recruit HP1a on TEs? One of the possibilities is based on the fact that the RNA helicase Rm62, which is a constituent of the Ago2 complex [25,63], interacts with histone methyltransferase Su(var)3-9 [64]. The latter enzyme is known to introduce H3K9me2/3 modifications in chromatin, thus recruiting HP1a to these sites [65,66,67].

**Figure 3:**
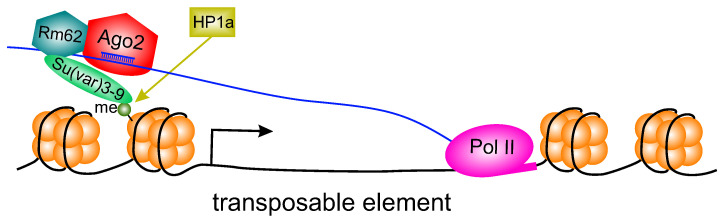
Hypothetical model of Ago2-mediated HP1a recruitment at TE chromatin. Ago2 transiently associates with nascent TE transcripts through complementarity with loaded siRNAs. The RNA helicase Rm62, a component of the Ago2 complex, recruits the histone methyltransferase Su(var)3–9, which introduces H3K9me2/3 into adjacent chromatin. HP1a binds to these histone modifications and co-transcriptionally represses TEs.

Based on our results and taking into account the data from the literature, we propose a hypothetical model of HP1a recruitment on TEs through the Ago2/siRNA complex (Figure 3). It should be noted, however, that some elements of this model have not been directly demonstrated and are only hypothesized by analogy with corresponding models in other organisms.

It has been shown that heterochromatin state is established during early embryonic development with the participation of the Piwi-dependent pathway and that it is somehow maintained in the non-gonadal somatic cells during development [68]. Importantly, the components of the piRNA-silencing system are not required at the later developmental stages to maintain heterochromatin state [68,69]. Furthermore, *piwi* mutations were shown to reduce only slightly, if at all, HP1a binding on TEs at the later stages [68,69]. Together with our results, these findings indicate that Piwi and Ago2 may both recruit HP1a on TEs in the non-gonadal somatic cells, with Ago2 exerting the stronger effect. This conclusion is in line with the results showing a dual-layer control of TE repression in *Drosophila* somatic cells by piRNA- and siRNA-silencing pathways [70]. The question then arises whether, similarly to Piwi, Ago2 recruits HP1a on TEs only in early embryos, or Ago2-mediated recruitment of HP1a occurs at the later developmental stages? We hypothesize that if Ago2 is able to recruit HP1a on TEs in early embryos and it is present in brain nuclei, then Ago2 may exert the same functions in the brain. Moreover, if, upon Lam-KD, Ago2 is indeed released from the association with the NL in neurons and thus recruits HP1a on TEs more efficiently, then the recruitment of HP1a via Ago2, at least in part, should take place in the brain.

In this study, we found that Ago2 is involved in the recruitment of HP1a on TEs, which is accompanied by a decrease in their chromatin accessibility. This is the hallmark of co-transcriptional TE silencing. However, post-transcriptional TE silencing may also operate. Future studies should clarify the contribution of co-transcriptional and post-transcriptional mechanisms in TE silencing in somatic *Drosophila* cells. Of note, the disturbance of silencing in the brain of *Ago2*-mutant flies results in only weak derepression of TEs, especially in comparison with strong TE derepression in *piwi*-mutant gonads. Yet, this weak TE derepression is sufficient to produce memory impairment and a shortened lifespan caused by increased transposition rates of TEs in fly neurons revealed upon aging [71]. Therefore, clarification of the details of these mechanisms is important in the context of known TE derepression in human neurodegenerative diseases [72].

## Data Availability

The original DamID-seq data presented in this study are openly available in the Gene Expression Omnibus (GEO) under accession number GSE304397. The scripts for analysis were deposited in the GitHub repository (https://github.com/LARG-IMG/Dm_brain_AGO2_2025 (accessed on 5 August 2025)). The link for the DamID profiles in the UCSC browser: (https://genome.ucsc.edu/s/Shevelyov/HP1a_DamID%20in%20brain%20upon%20Ago2mut%20or%20Lam%2DKD%20in%20neurons (accessed on 5 August 2025)).

## Supplementary Materials

The following supporting information can be downloaded at: https://www.mdpi.com/article/10.3390/cells14171361/s1. Figure S1: high specificity of the DamID procedure in the brain; Figure S2: replicates clustering; Figure S3: *Ago2* gene expression is significantly down-regulated in the *Ago2*-mutant brain; Figure S4: HP1a distribution is not notably changed in *Ago2^mut^* or Lam-KD brain; Table S1: NGS statistics; Table S2: changes in HP1a levels on TEs in *Ago2^mut^* or Lam-KD larval central brain; Table S3: primers for RT-qPCR.

## Abbreviations

The following abbreviations are used in this manuscript:

Ago2	Argonaute 2
ChIP	Chromatin immunoprecipitation
cHet	Constitutive heterochromatin
CPMs	Counts per million
DamID	Dam identification
fHet	Facultative heterochromatin
HP1a	Heterochromatin protein 1a
Lam-KD	Knock-down of lamin Dm0
LAD	Lamina-associated domain
LTR	Long terminal repeat
NL	Nuclear lamina
piRNA	Piwi-interacting RNA
RT-qPCR	Quantitative polymerase chain reaction after reverse transcription
siRNA	Small interfering RNA
TE	Transposable element
WT	Wild-type
