# Supplementary material for: Ago2-Mediated Recruitment of HP1a on Transposable Elements in Drosophila Brain
Source: Cells. 2025 Sep 1;14(17):1361. doi: 10.3390/cells14171361 (PMC12427935; doi:10.3390/cells14171361)
Supplement: Supplementary file 1 [file cells-14-01361-s001.zip › Supplementary Figures.pdf]

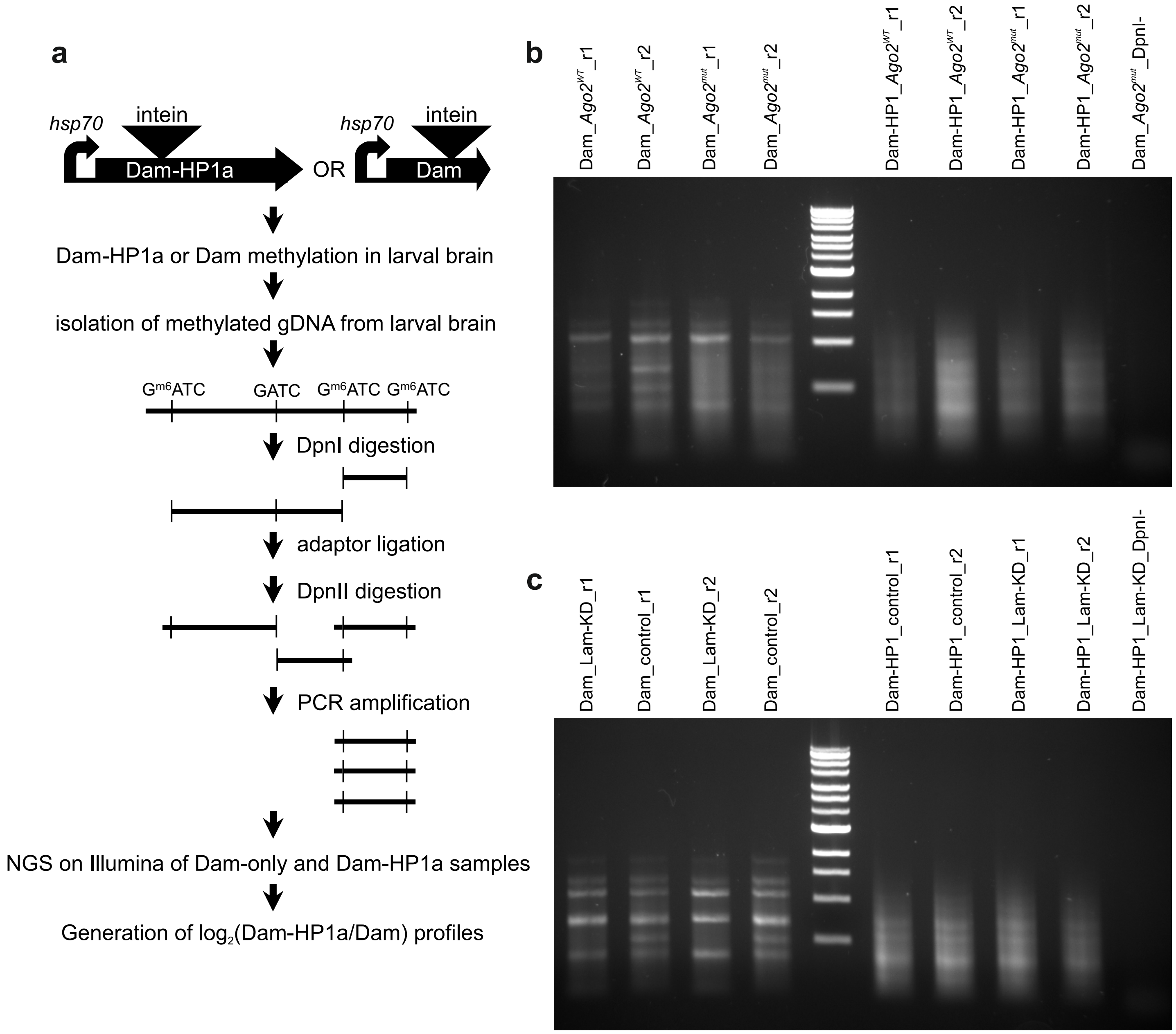

Supplementary Figure S1. High specificity of the DamID procedure in brain. (**a**) The principle of DamID procedure. (**b**,**c**) Gel-electrophoresis of PCR-amplified methylated genomic DNA fragments in *Ago2*-mutant brain (**b**), or in Lam-KD brain (**c**). The characteristic smear of amplified fragments is drastically less pronounced in the samples not treated by DpnI (DpnI-), thus indicating that adaptor ligation occurred on the fragments which were methylated *in vivo* by Dam-HP1a or Dam, and not on those formed as a result of random breaks in the genomic DNA during its isolation.

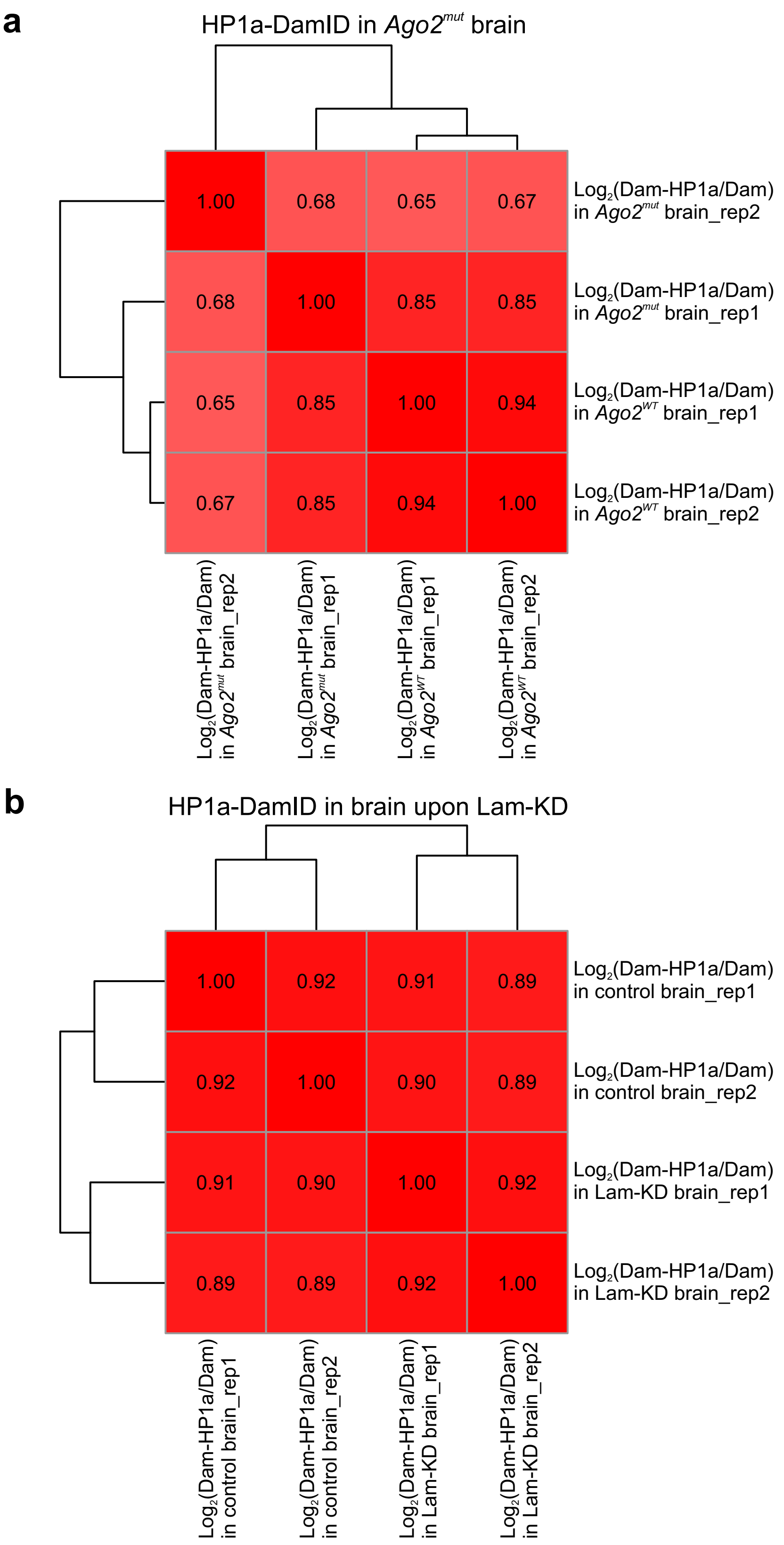

Supplementary Figure S2. Replicates clustering. (**a**,**b**) Dendrograms showing Pearson correlation coefficients between replicates for HP1a-DamID profiles in *Ago2*^{mut} and *Ago2*^{WT} (**a**) or Lam-KD and control (**b**) central brain.

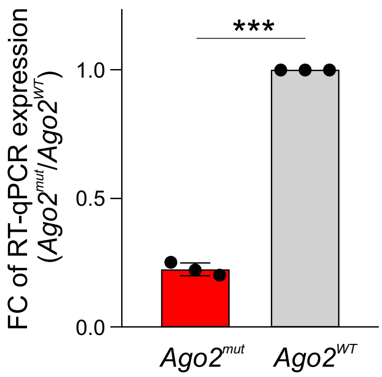

Supplementary Figure S3. *Ago2* gene expression is significantly down-regulated in *Ago2*-mutant brain. RT-qPCR analysis of *Ago2* expression in three replicates from the central brain of third instar larvae carrying $Ago2^{414}/Ago2^{454}$ trans-heterozygous mutant allels or $Ago2^{WT}$. Data were normalized on the *Actin5C* gene expression. *P*-value < 0.001 (***), one sample two tailed t-test.

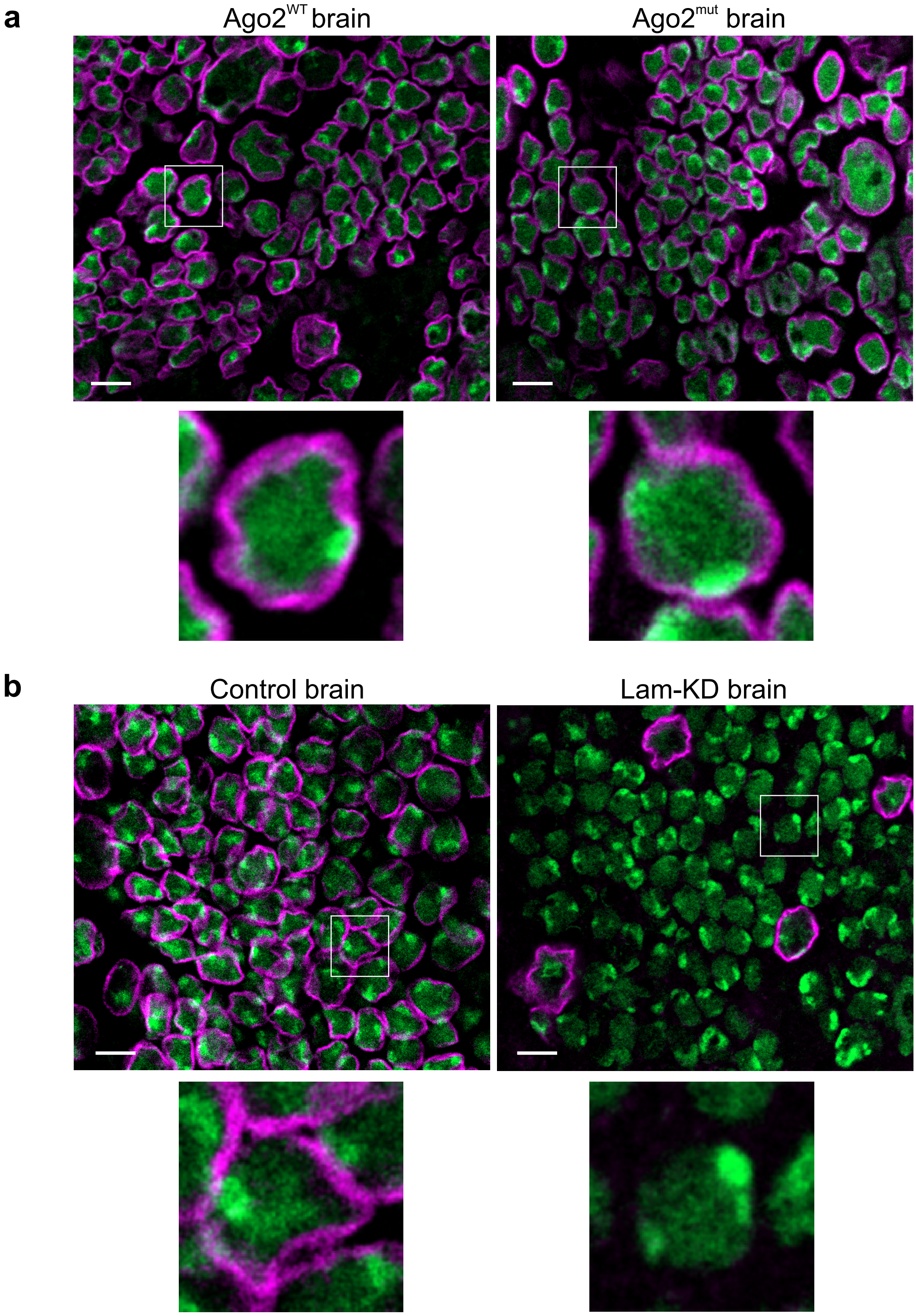

Supplementary Figure S4. HP1a distribution is not notably changed in *Ago2*^{mut} or Lam-KD brain. (**a**,**b**) Immunostaining of *Ago2*^{WT} and *Ago2*^{mut} (**a**) or control and Lam-KD (**b**) larval central brain with anti-HP1a (green) and anti-lamin Dm0 (violet) antibodies. Below is the enlarged images of nuclei outlined by white squares. Scale bars = 10 µm. Since knock-down of lamin *Dm0* gene was performed only in neurons, glial cells retain lamin staining.
